# The Novel Inhibitory Effect of YM976 on Adipocyte Differentiation

**DOI:** 10.3390/cells12020205

**Published:** 2023-01-04

**Authors:** Hee Jung Kim, Dong-Hoon Kim, Sung Hee Um

**Affiliations:** 1 Department of Molecular Cell Biology, Samsung Biomedical Research Institute, Sungkyunkwan University School of Medicine, Suwon 16419, Republic of Korea; 2 Department of Health Sciences and Technology, Samsung Advanced Institute for Health Sciences and Technology, Samsung Medical Center, Sungkyunkwan University, Seoul 06351, Republic of Korea; 3 Department of Pharmacology, Korea University College of Medicine, Seoul 02841, Republic of Korea; 4 Biomedical Institute for Convergence (BICS) at Sungkyunkwan University, Suwon 16419, Republic of Korea

**Keywords:** YM976, adipocyte differentiation, lipid metabolism, AMPK, obesity

## Abstract

The pyrimidine derivative YM976 (4-(3-chlorophenyl)-1,7-diethylpyrido(2,3-*d*)-pyrimidin-2(1*H*)-one) exerts anti-inflammatory and anti-asthmatic effects. Considering that accumulation of lipids in adipose tissue is accompanied by inflammation, we investigated whether YM976 affects adipocyte differentiation. We found that YM976 significantly decreased lipid accumulation without cytotoxicity and reduced the expression levels of peroxisome proliferator-activated receptor γ (PPARγ) and CCAAT/enhancer-binding protein α (C/EBPα) as well as their lipogenic regulators including fatty acid synthase (FASN) and fatty acid-binding protein 4 (FABP4) in 3T3-L1 cells induced for differentiation. YM976 mainly inhibited the early stage of adipocyte differentiation. Furthermore, intracellular cAMP level was elevated by YM976 resulting in increased phosphorylation of adenosine monophosphate-activated protein kinase (AMPK). Conversely, decreasing the levels of AMPK or treatment with Compound C, an AMPK inhibitor, lessened the suppressive effects of YM976 on PPARγ transcriptional activity and adipogenesis. Thus, our results suggest YM976 as a novel potential compound for controlling lipid accumulation and formation of adipocytes in obesity.

## 1. Introduction

Obesity is a global problem that is associated with numerous related diseases, including type 2 diabetes, dyslipidemia, cardiovascular disease, non-alcoholic fatty liver, and cancer [1]. Excessive accumulation of adipose tissue due to an imbalance in energy expenditure and intake leads to obesity [2]. Expansion of adipose tissue results from enlarged existing adipocytes and formation of new adipocytes [3]. Adipogenesis, also referred to as adipocyte hyperplasia is a process in which preadipocytes proliferate and differentiate into mature adipocytes; this process plays an essential role in obesity [4].

Adipocyte differentiation involves a complex step in which preadipocytes mature into lipid-bearing adipocytes [5]. This step requires stimulation by a hormone such as insulin, 1-methyl-3-isobutylxanthine (IBMX), or dexamethasone [6]. The process of adipocyte differentiation is classified into three stages of early, intermediate, and late [7]. Upon initiation of adipocyte differentiation, the expression of CCAAT/enhancer-binding protein β (C/EBPβ) and CCAAT/enhancer-binding protein δ (C/EBPδ) is increased and leads to expression of peroxisome proliferator-activated receptor γ (PPARγ) and CCAAT/enhancer-binding protein α (C/EBPα). Eventually, preadipocytes develop into mature adipocytes containing lipid droplets [8]. The process of adipocyte differentiation involves many adipogenic factors, including PPARγ, C/EBPs, fatty acid-binding protein 4 (FABP4), sterol regulatory element-binding protein-1 (SREBP1), and fatty acid synthase (FASN), and various signaling pathways including those of transforming growth factors-β (TGF-β), Wnt, Hedgehog, and AMP-activated protein kinase (AMPK) [9,10]. Identifying new target molecules and targeting agents that effectively control adipocyte differentiation may help establish new treatments for obesity and its relevant metabolic disorders.

Chronic inflammation is an almost universal condition in obesity [11]. In addition, it is associated with multiple pathophysiological conditions including heart disease, metabolic syndrome, and type 2 diabetes [12]. Previous studies have shown that levels of inflammatory mediators, including adipose tissue-derived inflammatory cytokines, were significantly increased in obesity as well as the associated metabolic diseases such as type 2 diabetes [12]. Modulation of inflammatory signaling in adipose tissue prevents the development of obesity in animal studies [11,13,14]. Consistently, adipocyte proliferation and differentiation triggers an inflammatory state that can be ameliorated by molecules and compounds that inhibit inflammation [15,16]. Thus, compounds with anti-inflammatory properties have emerged as novel anti-obesity agents.

YM976 (4-(3-chlorophenyl)-1,7-diethylpyrido(2,3-*d*)-pyrimidin-2(1*H*)-one) was synthesized based on a lead compound through random screening and exhibits anti-inflammatory activity with little emetogenic activity in several models such as allergic inflammation and experimental bladder inflammation [17]. Therefore, this compound is first proposed to reduce the emetic effect of compounds with anti-inflammatory activity [18]. Considering the close association between adipogenesis and inflammation [13], we hypothesized that YM976 suppresses adipocyte differentiation and adipogenic gene expression, accompanied by a reduction in intracellular lipid accumulation. We further analyzed how YM976 inhibits adipogenesis in 3T3-L1 cells.

## 2. Materials and Methods

### 2.1. Reagents

Penicillin-streptomycin (cat. LS 202-02), Trypsin-EDTA solution (cat. LS 015-08), and Dulbecco’s modified eagle medium (DMEM) (cat. LM 001-05) were purchased from WelGENE (Seoul, Republic of Korea). YM976 (cat. Y4877), Compound C (cat. 171260), Oil Red O (cat. O0625), dexamethasone (cat. D4902), dimethyl sulfoxide (DMSO, cat. D8418), IBMX (cat. I5879), 10% formalin (cat. HT501128), and insulin (cat. I0516) were purchased from Sigma-Aldrich (St. Louis, MO, USA). Antibodies against phospho-AMPK (cat. 2535), AMPKα (cat. 2532), C/EBPα (cat. 2295), FASN (cat. 3189), FABP4 (cat. 2120), phospho-CREB (cat. 9198), CREB (cat. 9197), phosphor-ACC (cat. 3661), and ACC (cat. 3662) were purchased from Cell Signaling Technology (Beverly, MA, USA). Antibodies against PPARγ (cat. sc-7273) and AMPKα siRNA (cat. sc-29674) were purchased from Santa Cruz Biotechnology (Santa Cruz, CA, USA). Fetal bovine serum (FBS, cat. 12483020), bovine serum (cat. 16170078), and ECL Western Blotting Substrate (cat. 32106) were purchased from Thermo Fisher Scientific (Waltham, MA, USA). Protease inhibitor cocktail (cat. 11697498001) was purchased from Roche Diagnostics (Indianapolis, IN, USA).

### 2.2. Cell Culture and Adipocyte Differentiation

We obtained 3T3-L1 preadipocytes (ATCC^®^ CL-173TM) from ATCC (Rockville, MD, USA) and cultured them in DMEM containing 10% bovine serum and 1% penicillin-streptomycin. To initiate adipocyte differentiation, confluent cells were incubated with DMEM including 10% FBS, 5.0 µg/mL insulin, 0.5 mM IBMX, and 1 µM dexamethasone for two days. This medium was replaced with a maintenance medium containing FBS and insulin after those two days and differentiated for six days. Cells were treated with DMSO (Veh, negative control) or YM976 from initiation to end of differentiation with every media change.

### 2.3. Oil Red O Staining

On day 6, fully differentiated 3T3-L1 cells were rinsed with phosphate-buffered saline (PBS), incubated with 10% formalin, and added to fresh formalin for more than 1 h. The cells were treated with 60% isopropanol for 5 min and then completely dried. Cells were stained with Oil Red O for at least 10 min and rinsed four times with distilled water. The stained cells were observed by microscopy, and images were obtained. For quantification of lipid accumulation, 100% isopropanol was added to cells, which were examined using a spectrophotometer at 500 nm [19].

### 2.4. MTT Assay

The cells were seeded onto 96-well plates and incubated in DMEM supplemented with 10% bovine serum for 24 h. Various concentrations of YM976 were used to treat cells for 24 h. Thereafter, MTT solution (5 mg/mL) was added, and cells were incubated for 4 h. The supernatant was removed and dissolved in DMSO, and absorbance was measured at 570 nm using a multi-well plate reader. 

### 2.5. RNA Isolation and Quantitative RT-PCR

Total RNA was extracted from 3T3-L1 cells using Trizol, following the manufacturer’s instructions. RNA (1 µg) was used to synthesize complementary DNA (cDNA) with random hexamer primers and SuperScript II reverse transcriptase. Trizol Reagent (cat. 15596026) and SuperScript II reverse transcriptase (cat. 18064022) were purchased from Thermo Fisher Scientific (Waltham, MA, USA). cDNA was used as a template for quantitative PCR using the QuantStudio 1 Real-Time PCR system (Applied Biosystems, Foster City, CA, USA) with SYBR green [20] and specific primers (Table 1).

### 2.6. Protein Extraction and Immunoblotting

Protein extraction and immunoblotting were performed as described previously [21]. Cells were washed with ice-cold PBS and lysed in RIPA buffer with 0.1 M NaF, 0.25% Na deoxycholate, and 2 mM Na_3_VO_4_ for at least 20 min on ice and then centrifuged to collect lysate. Protein concentrations were calculated using the Bio-Rad protein assay reagents. Proteins were separated by SDS-PAGE and transferred to a PVDF membrane. The membranes were blocked in bovine serum albumin (5%) for 1 h. The membranes were then incubated with primary antibodies against phospho-AMPK, AMPKα, C/EBPα, FASN, FABP4, phospho-CREB, CREB, phospho-ACC, ACC, and PPARγ overnight at 4℃. After washing three times in TBS-T, the membranes were incubated with goat anti-rabbit or goat anti-mouse horseradish peroxidase-conjugated IgG secondary antibodies. Protein signals were developed using the ECL system according to the manufacturer’s protocol. 

### 2.7. AMPK α Silencing

For siRNA transfection, 3T3-L1 cells were seeded in six-well plates. After 24 h, cells were transfected with non-silencing RNA (si-NS) or AMPKα siRNA (si-AMPKα, cat. sc-45413) using Lipofectamine RNAiMAX Transfection Reagent (cat. 13778075) purchased from Thermo Fisher Scientific (Waltham, MA, USA), following the manufacturer’s procedures.

### 2.8. Measurement of Intracellular Cyclic Adenosine Monophosphate (cAMP) 

The intracellular cAMP level in 3T3-L1 cells was evaluated using a cAMP direct immunoassay (cat. ab65355) purchased from Abcam (Boston, MA, USA), following the manufacturer’s protocol. After treatment with vehicle or YM976, cell lysates were transferred to 96-well plates pre-coated with cAMP polyclonal antibodies. A cAMP-horseradish peroxidase (HRP) conjugate was used to directly compete for binding to cAMP antibodies. After plates were incubated and washed, HRP activity was measured using a plate reader.

### 2.9. Luciferase Reporter Assay

Cells were seeded into six-well plates and transfected with PPARγ expression plasmid and empty vector (pGL3-Luc) or the PPRE expression vector (pPPRE-Luc) [22] using FuGENE9 Transfection reagent (cat. 6365809001), purchased from Roche Diagnostics (Indianapolis, IN, USA). After 24 h, the cells were treated with Veh or YM976 in the presence of differentiation media. Luciferase activity was measured using a Luciferase Assay kit (cat. E1500), purchased from Promega (Madison, WI, USA), following the manufacturer’s instructions.

### 2.10. Statistics

Data are shown as mean ± standard deviation (SD). Statistical analyses were performed with Prism 9.0 software using one-way analysis of variance (ANOVA) followed by post hoc comparison test (Dunnett’s or Tukey’s test) or Student’s *t* test. Differences with *p* values < 0.05 were regarded as statistically significant.

## 3. Results

### 3.1. Inhibitory Effect of YM976 on Lipid Accumulation in 3T3-L1 Cells

**Figure 1:**
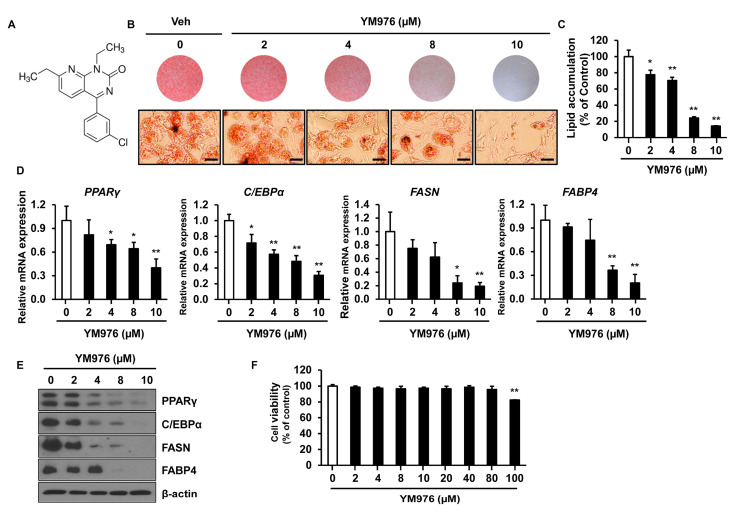
YM976 inhibits adipocyte differentiation. (**A**) The chemical structure of YM976. (**B**) 3T3-L1 cells were induced to differentiate for six days and treated with Veh or various concentrations of YM976. Microscopic image after staining with Oil Red O. Scale bar = 100 µm. (**C**) Quantification of Oil Red O staining by spectrophotometry. (**D**) The mRNA expression of *PPARγ, C/EBPα, FASN,* and *FABP4* in cells after treatment with various concentrations of YM976 was analyzed by qRT-PCR. (**E**) Protein levels of PPARγ, C/EBPα, and C/EBPβ were analyzed by Western blot. (**F**) The effect of YM976 on cell viability. In (**C**–**E**), *n* = 3 per group. Images and blots are representative of three independent experiments. Values indicate mean ± SD. * *p* < 0.05; ** *p* < 0.01. Data were assessed by one-way ANOVA with Dunnett’s post hoc test.

The chemical structure of YM976 is presented in Figure 1A [18]. To determine whether YM976 affects adipocyte differentiation, 3T3-L1 preadipocytes were induced for differentiation and treated with vehicle (Veh) or various concentrations of YM976 for six days. Differentiated cells were examined by staining with Oil Red O solution and quantification of triglyceride content in adipocytes. Oil Red O staining showed reduced size and number of lipid droplets in cells treated with 8 and 10 µM of YM976 compared with Veh-treated cells (Figure 1B). In cells treated with 2 to 10 µM YM976, inhibition of lipid accumulation was observed, ranging from 23% to 86% compared with Veh treatment (Figure 1C). We next investigated whether the inhibitory effect of YM976 on lipid accumulation influences the expression of C/EBPα, PPARγ, FASN, and FABP4, which have essential roles in adipocyte differentiation. The mRNA expression of C/EBPα, PPARγ, FASN, and FABP4 decreased greatly after treatment with 10 µM YM976 (Figure 1D). Consistent with these results, the protein levels of C/EBPα, PPARγ, FASN, and FABP4 were reduced markedly following treatment with 10 µM YM976 (Figure 1E). In addition, we assessed the effects of YM976 on lipid accumulation and mRNA expression levels of adipogenic transcription factors in undifferentiated cells as a negative control. Cells were treated with Veh or 10 μM YM976 at initiation of differentiation for two days and further cultured in maintenance medium for six days. Medium was changed every second day for six days in the presence of Veh or 10 μM YM976. The levels of lipid accumulation and adipogenic gene expression were reduced by YM976 treatment (Supplementary Figure S1). To examine whether cell viability was affected by YM976, we performed an MTT assay. YM976 did not show cytotoxicity up to a concentration of 80 µM in 3T3-L1 cells (Figure 1F). Cell viability was reduced at a concentration of 100 µM or higher. Taken together, these results suggest that 10 µM of YM976 strongly inhibits lipid accumulation and adipocyte differentiation without cytotoxicity. 

### 3.2. YM976 Suppresses the Early Stage of Adipocyte Differentiation

**Figure 2:**
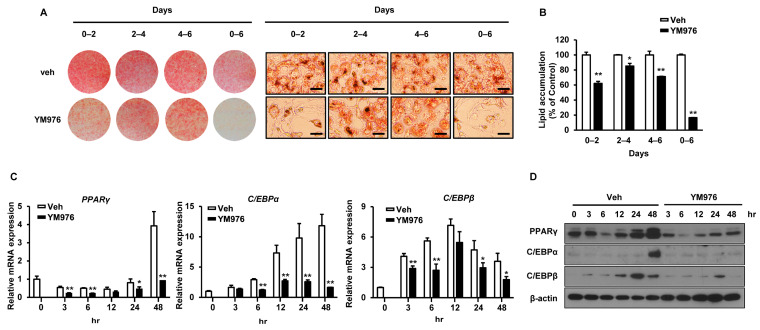
The inhibitory effect of YM976 occurs in the early stage of adipocyte differentiation. 3T3-L1 cells were induced for differentiation and then treated with Veh or 10 µM of YM976 at the indicated time points. (**A**) Cells were stained with Oil Red O and analyzed by microscopy. Scale bar = 100 µm. (**B**) Quantification of Oil Red O staining by spectrophotometry. (**C**) The mRNA expression of *PPARγ, C/EBPα,* and *C/EBPβ* was analyzed by qRT-PCR. (**D**) Protein levels of PPARγ, C/EBPα, and C/EBPβ were analyzed by Western blot. In (**B**,**C**), *n* = 3 per group. Images and blots are representative of three independent experiments. Values indicate mean ± SD. * *p* < 0.05; ** *p* < 0.01. Data were assessed using Student’s *t*-test.

Adipocyte differentiation progresses in three stages of early, intermediate, and late [23]. To determine which stage was most influenced by YM976, cells induced for differentiation were treated with 10 µM YM976 on specific days corresponding to differentiation stage: early: days 0–2, intermediate: days 2–4, late: days 4–6, and overall: days 0–6. While Oil Red O staining revealed a significant decrease in adipocyte differentiation in all periods, the greatest inhibitory effect of YM976 was observed at the initial and overall stages (Figure 2A). YM976 exerted a stronger inhibitory effect on lipid accumulation at the early stage compared with the intermediate or late stage (Figure 2B), suggesting that YM976 acts mainly at the early stage of differentiation. In addition, YM976 significantly reduced the mRNA expression levels of *PPARγ, C/EBPβ,* and *C/EBPα* for 3 h after the start of adipocyte differentiation (Figure 2C). Consistent with this result, the protein level of PPARγ was significantly decreased after 3 h following YM976 treatment, while the protein level of C/EBPα was reduced after 48 h of YM976 treatment (Figure 2D). Collectively, these results indicate that YM976 represses the early stages of adipocyte differentiation by decreasing the mRNA and protein levels of C/EBPα and PPARγ. 

### 3.3. YM976 Induces Intracellular cAMP Level and Increases Phosphorylation of PKA and AMPK

**Figure 3:**
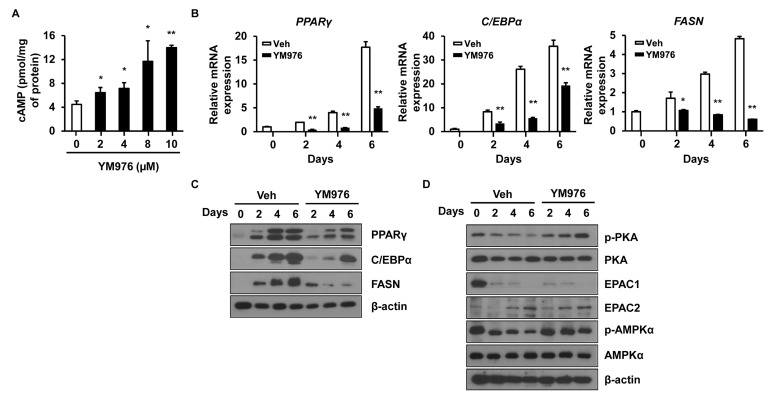
YM976 increases the level of cAMP and decreases the expression of adipogenic genes. Cells were treated with Veh or YM976 at the indicated days. (**A**) Intracellular cAMP level was analyzed by ELISA. (**B**) The mRNA expression levels of *PPARγ, C/EBPα,* and *FASN* after treatment with YM976 were determined by qRT-PCR. (**C**) Protein levels of PPARγ, C/EBPα, and FASN were analyzed by Western blot. (**D**) Expression levels of indicated protein were assessed by immunoblotting. In (**A**,**B**), *n* = 3 per group. In (**C**,**D**), blots are representative of three independent experiments. Values indicate mean ± SD. * *p* < 0.05; ** *p* < 0.01. Data were assessed by one-way ANOVA with Dunnett’s post hoc test.

A high level of intracellular cAMP inhibits adipocyte differentiation by decreasing the expression of PPARγ and C/EBPα in adipocytes [24]. To examine whether YM976 affects cAMP level, intracellular cAMP level was analyzed after treatment with YM976 for 1 h. YM976 from 2 µM to 10 µM increased intracellular cAMP level compared with Veh-treatment (Figure 3A). 

Various signaling pathways affect the early stages of adipocyte differentiation, such as extracellular signal-regulated kinase 1/2 (ERK1/2), mitogen-activated protein kinase, AKT, protein kinase A (PKA), and AMP-activated protein kinase (AMPK) signaling [9,25]. PKA and AMPK kinases are phosphorylated with increasing cAMP level and inhibit the early stages of adipocyte differentiation [24,26]. To determine which signaling molecule is related to the anti-adipogenic effect of YM976, cells were treated with YM976 and harvested on specific days. The mRNA expression of adipogenic transcription factors including *PPARγ, C/EBPα,* and *FASN* in YM976-treated cells was decreased significantly compared with levels in Veh-treated cells on days 2, 4, and 6 (Figure 3B). The protein levels of PPARγ, C/EBPα, and FASN were also strongly reduced in YM976-treated cells compared with Veh-treated cells on days 2, 4, and 6 (Figure 3C). Treatment with YM976 markedly increased phosphorylation levels of PKA and its downstream targets, AMPK, and cyclic-AMP response element-binding protein (CREB) (Figure 3D). Another target affected by cAMP is exchange proteins directly activated by cAMP (EPACs), which act as a crucial regulator in glucose homeostasis and energy balance [27]. However, YM976 did not influence the protein levels of EPAC1 and EPAC2. Together, these results indicate that YM976 decreases the expression of adipogenic genes and induces increased phosphorylation levels of AMPK and PKA during adipocyte differentiation. 

### 3.4. Depletion of AMPKα Partially Reverses the Inhibitory Effect of YM976 on Adipocyte Differentiation

**Figure 4:**
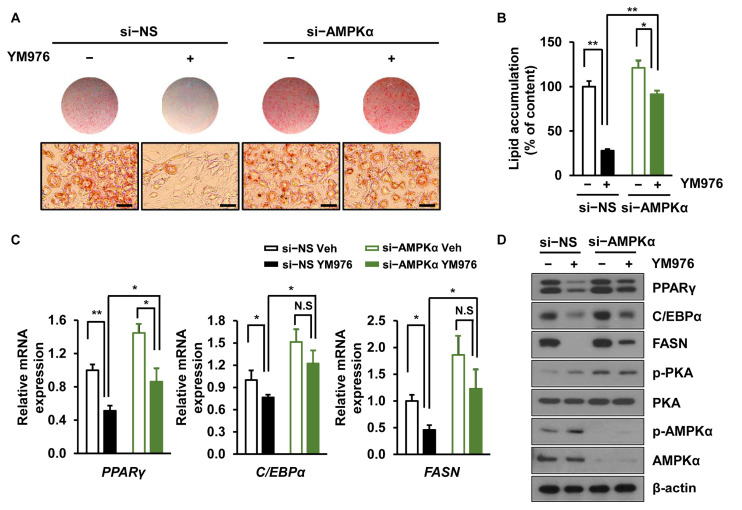
AMPKα depletion attenuates the inhibitory effect of YM976 on adipocyte differentiation. Cells were transfected with si-NS or si-AMPKα and then treated with Veh or 10 µM YM976. (**A**) Cells were stained with Oil Red O and observed with microscopy. Scale bar = 100 µm. (**B**) Quantification of Oil Red O staining by spectrophotometry. (**C**) The mRNA expression levels of *PPARγ, C/EBPα,* and *FASN* were examined by qRT-PCR. (**D**) Expression levels of indicated protein were assessed by immunoblotting. In (**B**,**C**), *n* = 3 per group. In (**A**,**D**), images and blots are representative of three independent experiments. Values indicate mean ± SD. * *p* < 0.05; ** *p* < 0.01. N.S., not significant. Data were assessed by one-way ANOVA with Tukey’s post hoc test.

AMPKα, one of the main regulators of cellular energy balance, is activated by its phosphorylation at Thr172, leading to inhibition of lipogenesis and adipogenesis [28]. Considering our result showing that phosphorylation of AMPKα is increased by YM976 (Figure 3D), we next investigated whether depletion of AMPKα can reverse the anti-adipogenic effect of YM976. Cells were transfected with si-NS or si-AMPKα and treated with Veh or YM976 after induction of adipocyte differentiation. The anti-adipogenic effect of YM976 decreased in AMPKα depleted cells (Figure 4A), and lipid accumulation was increased in AMPKα knockdown cells upon treatment with YM976 (Figure 4B). Additionally, reduced mRNA expression of *FASN, PPARγ*, and *C/EBPα* by YM976 treatment was restored after AMPKα depletion (Figure 4C). Protein levels of FASN, C/EBPα, and PPARγ partially recovered in AMPKα-depleted cells after treatment with YM976 (Figure 4D). Collectively, these results suggest that YM976 suppresses adipocyte differentiation by inhibiting the expression of adipogenic transcription factors via AMPKα. 

### 3.5. AMPK Inhibitor (Compound C) Attenuates the Inhibitory Effect of YM976 on Adipocyte Differentiation 

**Figure 5:**
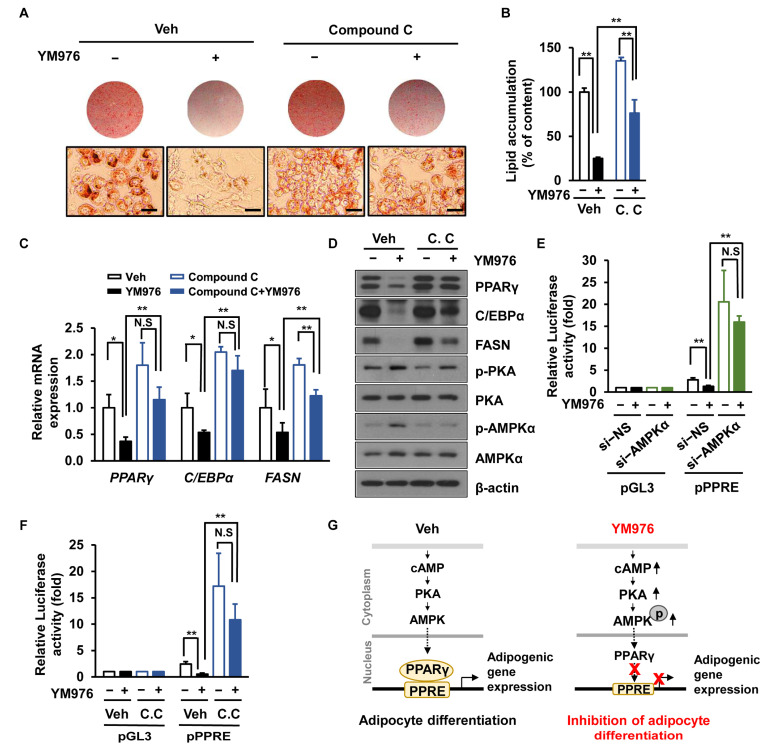
AMPK inhibitor lessens the inhibitory effect of YM976 on adipocyte differentiation. (**A**–**D**) Cells were pre-treated with Compound C (1 µM) for 1 h and then incubated with YM976 during adipocyte differentiation for six days. (**A**) Cells were stained with Oil Red O and observed by microscopy. Scale bar = 100 µm. (**B**) Quantification of Oil Red O staining by spectrophotometry. (**C**) The mRNA expression levels of *PPARγ, C/EBPα,* and *FASN* were examined by qRT-PCR. (**D**) Levels of indicated proteins were analyzed by immunoblotting. (**E**) After silencing with si-NS or si-AMPKα, 3T3-L1 cells were transfected with PPARγ expression plasmid and pGL3 luciferase vector (pGL3) or PPAR-response element-binding luciferase vector (PPRE-Luc) for 24 h. Cells were then treated with Veh or YM976 for 24 h. Luciferase assay was performed to measure the transcription activity of PPARγ. (**F**) After pre-incubation with Veh or Compound C (1 µM) for 1 h, 3T3-L1 cells were transfected with PPARγ expression plasmid and pGL3 or PPRE-Luc or for 24 h. Cells were then treated with Veh or YM976 for 24 h. Luciferase assay was performed to measure the transcriptional activity of PPARγ. (**G**) Proposed model of YM976 inhibition of adipocyte differentiation. YM976 increases the levels of cAMP and AMPK phosphorylation to suppress the transcriptional expression of adipogenic genes, resultings in reduced adipogenesis. pGL3, pGL3 luciferase vector; pPPRE, peroxisome proliferator response element reporter plasmid; C.C., Compound C; N.S., not significant. In (**B**,**C**,**E**,**F**), *n* = 3 per group. In A and D, images and blots are representative of three independent experiments. Values indicate mean ± SD. * *p* < 0.05; ** *p* < 0.01. Data were assessed by one-way ANOVA with Tukey’s post hoc test.

Compound C blocks the activity of AMPK, leading to increased lipid storage and synthesis of fatty acids and triglycerides in 3T3-L1 cells [29]. We assessed whether Compound C affects adipocyte differentiation upon treatment with YM976. The anti-adipogenic effect of YM976 was reversed by treatment with Compound C (Figure 5A). The intracellular triglyceride content was also restored in Compound C-treated cells following YM976 treatment (Figure 5B). Consistent with these results, decreased mRNA expression of *FASN, PPARγ,* and *C/EBPα* by YM976 was restored after Compound C treatment (Figure 5C). Additionally, protein levels of PPARγ and C/EBPα were recovered in cells treated with Compound C and YM976 (Figure 5D). 

AICAR and metformin, AMPK activators, inhibit the transcriptional activity of PPARγ in cells, whereas Compound C (an AMPK inhibitor) increases PPARγ transactivation [30]. Thus, we next examined whether AMPK depletion affects the transcriptional activity of PPARγ upon treatment with YM976. We performed luciferase assays by first transfecting cells with si-NS or si-AMPKα and then with PPARγ expression plasmid and pGL3 luciferase vector (pGL3-Luc) or PPAR-response element-binding luciferase vector (PPRE-Luc). Cells were treated with Veh or YM976 for another 24 h, and PPARγ activity was measured using a luciferase assay. These results showed that YM976 inhibited the transcriptional activity of PPARγ; conversely, depletion of AMPKα restored the transcriptional activity of PPARγ (Figure 5E). Furthermore, when cells were treated with Compound C, the transcriptional activity of PPARγ was recovered under YM976 treatment (Figure 5F), suggesting that depletion of AMPK or treatment with Compound C restored the transcriptional activity of PPARγ in YM976-treated cells. Collectively, our results indicate that YM976 inhibits the transcriptional activity of PPARγ via AMPK, leading to the suppression of adipocyte differentiation (Figure 5G). 

## 4. Discussion

Obesity is related to chronic low-grade inflammation and increases the risk for many metabolic diseases such as cardiovascular disease, type 2 diabetes, and chronic kidney disease [11,12,13,14,31]. Chronic inflammation contributes to the development of obesity and insulin resistance as well as the differentiation of pre-adipocytes to mature adipocytes [32]. Therefore, compounds or agents exhibiting anti-inflammatory properties have been proposed for the treatment of obesity [15]. YM976 is a pyrimidine derivative and possesses anti-inflammatory, anti-asthmatic, and bronchodilating effects with low emetogenic activity [33]. Given the anti-inflammatory action of YM976, its effect on adipocyte differentiation had not been fully explored. Here, we found that YM976 exhibits a novel effect in suppressing adipocyte differentiation.

Adipocyte differentiation is one of the critical steps for development of obesity because excessive fat accumulation occurs from an increase in number and/or size of differentiated mature adipocytes [6]. This process is controlled by several transcription factors including PPARγ, C/EBPα, C/EBPβ, and C/EBPδ, which induce the expression of lipogenic genes such as *FASN, FABP4*, and *SREBP1* [7]. Our results suggest that YM976 reduces lipid accumulation by decreasing the expression of adipogenic transcription factors including C/EBPα and PPARγ as well as lipogenic factors such as FABP4 and FASN during early adipocyte differentiation. 

cAMP is an essential mediator of various responses including cell proliferation, apoptosis, and differentiation [34]. Elevated cAMP level increases the phosphorylation of AMPK, leading to inhibition of adipocyte differentiation [24,35]. Our results revealed that YM976 increased intracellular cAMP level and phosphorylation of AMPK and PKA, resulting in decreased expression of PPARγ and C/EBPα. Similarly, piperonal, an aromatic compound, inhibits adipogenesis by inducing high intracellular cAMP level and resulting in phosphorylation of AMPK [36]. Filbertone, a compound derived from hazelnuts, prevented body weight gain in HFD-fed mice by increasing the cAMP level and activation of PKA and AMPK in adipose tissue [37]. Various stimulators for the cAMP pathway, such as 8-bromo-cAMP and forskolin suppress the differentiation of preadipocytes [24,38]. Thus, our results suggest that YM976 may increase the level of cAMP, activating PKA-AMPK signaling, which eventually inhibits adipocyte differentiation. On the other hand, YM976 exhibits a suppressive effect on PDE4 activity, which increases cAMP level in peripheral blood mononuclear cells [39]. Whether the anti-adipogenic effect of YM976 is regulated by PDE4 would be interesting to explore in future studies. 

AMPK is one of the main regulators of metabolism and is an essential target in the treatment of obesity and type 2 diabetes [40]. Activation of AMPK exerts an inhibitory effect on preadipocyte differentiation by decreasing the expression of C/EBPα and PPARγ [9,41]. Our results showed that YM976 increased the phosphorylation of AMPK, decreasing the expression of adipogenic genes, whereas AMPK silencing or Compound C treatment reversed the effects of YM976. Similarly, metformin and A-769662, AMPK activators, appear to inhibit lipid accumulation by decreasing the mRNA level of PPARγ through activation of AMPK in the early stage of adipocyte differentiation [42]. The small-molecule COH-SR4 (mitochondria uncoupler SR4), an AMPK activator, inhibits adipocyte differentiation and proliferation at the concentration of 100 µM, whereas its effect is restored upon AMPKα1/α2 silencing in COH-SR4 treated cells [43]. Notably, YM976 exerted an anti-adipogenic effect at a much lower concentration of 10 µM compared to COH-SR4 or metformin at concentrations of 100 µM or 500 µM. Therefore, YM976 is likely to be more effective in inhibiting adipocyte differentiation via AMPK activation at a lower concentration supporting its benefit in pharmacological dosage. We further showed that AMPK inhibition via treatment with an AMPK inhibitor, Compound C, or AMPK silencing recovered the YM976-induced suppression of the transcriptional activity of PPARγ in 3T3-L1 cells. Given the strong inhibitory action of YM976 on adipogenesis, it would be of interest to determine whether YM976 is effective in controlling fat accumulation and lipid metabolism in adipose tissue.

In conclusion, our study demonstrated that YM976 increased cAMP level and phosphorylation of AMPK, suppressing the transcriptional activity of PPARγ and resulting in inhibition of adipocyte differentiation. These results indicate that the development of pyrimidine derivatives targeting AMPK may be a potential therapeutic strategy to prevent obesity.

## Figures and Tables

**Table 1:** List of primers used for quantitative RT-PCR.

Name	Forward (5′→3′)	Reverse (5′→3′)
*PPARγ*	GGGTGAAACTCTGGGAGATTCTCC	CAGCAACCATTGGGTCAGCTCT
*C/EBPα*	ACAACATCGCGGTGCGCAAGA	TGCCATGGCCTTGACCAAGGAG
*FASN*	CGGAAACTGCAGGAGCTGTC	CACGGAGTTGAGCCGCAT
*C/EBPβ*	GTCCAAACCAACCGCACAT	CAGAGGGAGAAGCAGAGAGTT
*FABP4*	TGG AAG CTT GTC TCC AGT GA	AAT CCC CAT TTA CGC TGA TG
*GAPDH*	GTCTTCCTGGGCAAGCAGTA	CTGGACAGAAACCCCACTTC

## Data Availability

Not applicable.

## Supplementary Materials

The following supporting information can be downloaded at: https://www.mdpi.com/article/10.3390/cells12020205/s1, Figure S1: YM976 suppresses adipocyte differentiation.
