# Supplementary material for: The Novel Inhibitory Effect of YM976 on Adipocyte Differentiation
Source: Cells. 2023 Jan 4;12(2):205. doi: 10.3390/cells12020205 (PMC9856710; doi:10.3390/cells12020205)
Supplement: Supplementary file 1 [file cells-12-00205-s001.zip › cells-1956957-supplementary.pdf]

Supplementary Figure

The novel inhibitory effect of YM976 on adipocyte differentiation

Hee Jung Kim [1,2], Dong-Hoon Kim [3*] and Sung Hee Um [1, 2, 4*]

[1] Department of Molecular Cell Biology, Samsung Biomedical Research Institute, Sungkyunkwan University School of Medicine, Suwon 16419, Republic of Korea

[2] Department of Health Sciences and Technology, Samsung Advanced Institute for Health Sciences and Technology, Samsung Medical Center, Sungkyunkwan University, Seoul 06351, Republic of Korea

[3] Department of Pharmacology, Korea University College of Medicine, Seoul 02841, Republic of Korea

[4] Biomedical Institute Convergence at Sungkyunkwan University, Suwon 16419, Republic of Korea

* Correspondence: LDHKIM@korea.ac.kr (D.H.K.); shum@skku.edu (S.H.U.)

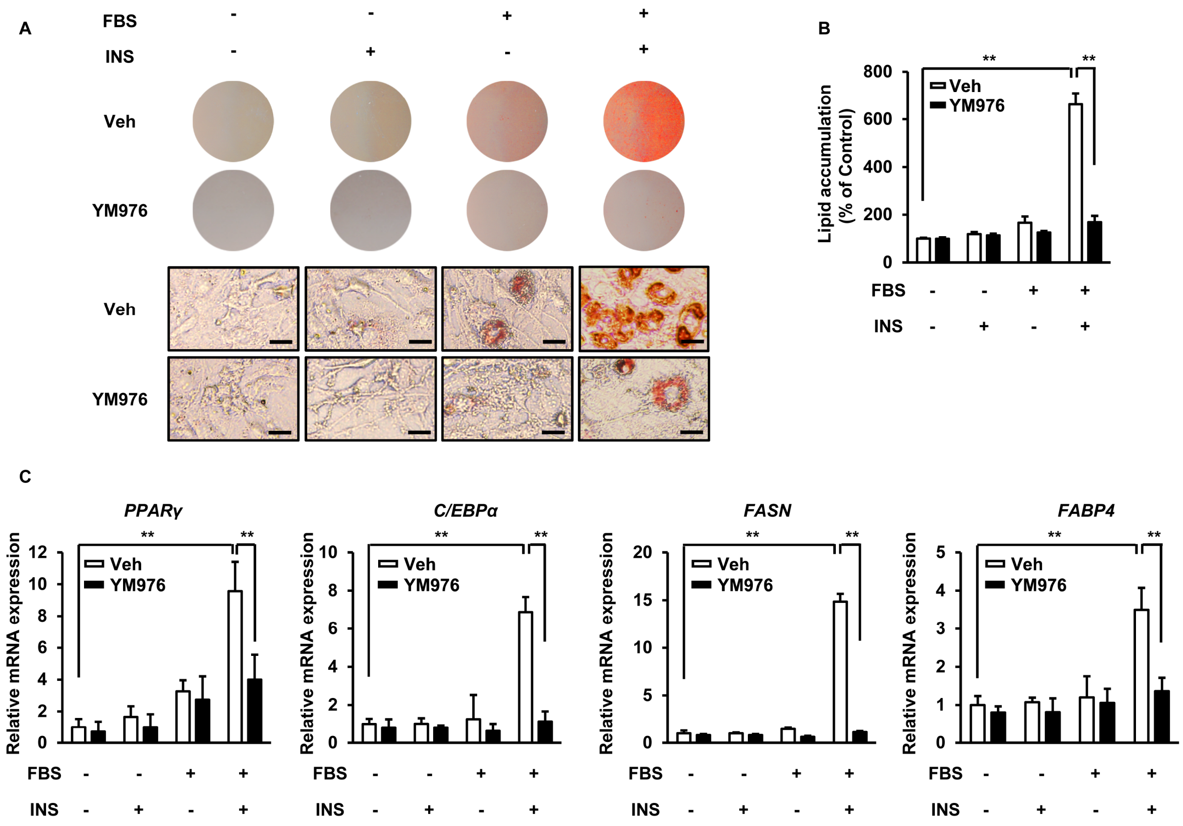

Figure S1. YM976 suppresses adipocyte differentiation. 3T3-L1 cells were exposed to differentiation media in the presence or absence of FBS or insulin and treated with Veh or 10 μM YM976 every other day from initiation to six days of differentiation. (A) Microscopic images of Oil Red O Staining. (B) Quantification of Oil Red O staining by spectrophotometry. (C) The mRNA expression of PPARγ, C/EBPα, FASN, and FABP4 in cells treated with Veh or YM976 was analyzed by qRT-PCR. In B-C, n = 3 per group. Images are representative of three independent experiments. Scale bar = 100 μm. Values indicate mean ± SD. ***p* < 0.01. Data were assessed by one-way ANOVA.
